# The role of T-cells in head and neck squamous cell carcinoma: From immunity to immunotherapy

**DOI:** 10.3389/fonc.2022.1021609

**Published:** 2022-10-20

**Authors:** Marcos Paulo S. Damasio, Camila Sales Nascimento, Lidia M. Andrade, Vivian L. de Oliveira, Carlos Eduardo Calzavara-Silva

**Affiliations:** ^1^ Division of Gastroenterology, Massachusetts General Hospital and Harvard Medical School, Boston, MA, United States; ^2^ Grupo de pesquisa em Imunologia Celular e Molecular, Fundação Oswaldo Cruz, Instituto Rene Rachou, Belo Horizonte, MG, Brazil; ^3^ Departamento de Genética, Ecologia e Evolução, Departamento de Física, Nanobiomedical Research Group, Universidade Federal de Minas Gerais, Belo Horizonte, MG, Brazil; ^4^ Universidade Federal do ABC, Centro de Ciências Naturais e Humanas, São Paulo, Brazil; ^5^ Laboratório de Imunologia, LIM19, Instituto do Coração (InCor), Hospital das Clínicas da Faculdade de Medicina da Universidade de São Paulo (HCFMUSP), São Paulo, Brazil

**Keywords:** T-cell, HNSCC, tumor immune evasion, checkpoint inhibitors, immunotherapy, nanomedicine

## Abstract

Head and neck squamous cell carcinoma (HNSCC) encompass a group of complex entities of tumours affecting the aerodigestive upper tract. The main risk factors are strongly related to tobacco and alcohol consumption, but also HPV infection is often associated. Surgery, radiotherapy and/or chemotherapy are the standard treatments, though the 5-year overall survival is less than 50%. The advances in genomics, molecular medicine, immunology, and nanotechnology have shed a light on tumour biology which helps clinical researchers to obtain more efficacious and less toxic therapies. Head and neck tumours possess different immune escape mechanisms including diminishing the immune response through modulating immune checkpoints, in addition to the recruitment and differentiation of suppressive immune cells. The insights into the HNSCC biology and its strong interaction with the tumour microenvironment highlights the role of immunomodulating agents. Recently, the knowledge of the immunological features of these tumours has paved the way for the discovery of effective biomarkers that allow a better selection of patients with odds of improving overall survival through immunotherapy. Specially biomarkers regarding immune checkpoint inhibitors antibodies, such as anti-PD-1/PD-L1 and anti-CTLA-4 in combination with standard therapy or as monotherapy. New immunotherapies to treat head and neck cancer carcinomas, such as CAR T cells and nanoparticles have been the center of attention and in this review, we discuss the necessity of finding targets for the T cell in the cancer cells to generate CAR T cells, but also the relevance of evaluating specificity and safety of those therapies.

## Introduction

Among epithelial cancers, head and neck squamous cell carcinoma (HNSCC) is the 6^th^ most prevalent tumour presenting more than 650.000 cases and 330.000 deaths annually worldwide (1). The most frequent sites affected are oral cavity, oropharynx, nasopharynx, larynx, lips, sinuses, and upper oesophagus. The etiological factors correlated to these tumours include tobacco use, alcohol consumption, human papillomavirus (HPV) infection mainly for oropharyngeal cancer, in younger patients, and Epstein-Barr virus (EBV) infection. Although improvements in treatment modalities, including immunotherapy, have demonstrated good results, the overall survival has not increased significantly over the past few years. In general, treatment failure is represented by local and regional recurrences. Although less frequent, distant metastasis (DM) has also been reported and occurs in 10% to 24% of all HNSCC cases, affecting primarily the lungs, bones and liver (2, 3). Patients with early-stage tumour (I and II) have 60–95% possibility of successful treatment, but a significant proportion of patients initially diagnosed with locoregionally advanced HNSCC develop disease recurrence, in 30% to 45% within the first year following multi-modal treatment consisting of surgery and/or chemoradiation (2, 4, 5).

For the last decades, many researchers have been investigating new approaches for the development of biomarker-based treatments that can guide physicians to decisions regarding patient`s outcomes especially in recurrent/metastatic diseases. The discovery and development of specific molecular targets have demonstrated therapeutic potential in cancer treatment, based on signal transduction alterations in cancer cells (6), mostly applying monoclonal antibodies or tyrosine kinase inhibitors that target specific receptors correlated to proliferative pathways like the epidermal growth factor receptor (EGFR) and angiogenesis characterized by the vascular endothelial growth factor receptor (VEGFR) (7).

A deeper understanding of the role of the immune system in cancer progression has provided knowledge of the mechanisms behind cancer immunosurveillance evasion. An enhanced immune response can lead to resistant tumor formation due to a process of immunoediting and the presence of immune suppressive factors in the tumor microenvironment (TME) may play an important role in the tumor’s growth. It is already accepted that the composition and frequency of immune cells within the TME and peripheral blood are closely related with tumorigenesis (8). Evidences of a decreased immunogenicity associated with a heightened immune dysfunction in HNSCC has been observed, which suggest a negative impact on the outcome and prognosis of these patients (9).

Distinct cell types and molecules such as cytokines and chemokines contribute to the immune response coordinated to target tumor cells in head and neck cancer. The presence of immune cells, primarily dendritic cells, T-lymphocytes, B cells and plasma cells, some natural killer cells (NK), macrophages and eosinophils impact the onset and progression of HNSCC (10). However, head and neck tumors can establish an immunosuppressive microenvironment based on the mutual interactions between the tumor and its host. These tumors develop different mechanisms to escape from the immune surveillance system that involve the direct inhibition of T-cells through soluble or surface molecules leading to the recruitment of suppressive cell populations. The escape of the tumor-associated antigens (TAA) from host immunity indicates a failure of the immune system to control tumor progression (11).

Several strategies underlying tumor immune scape including the modulation of inflammatory cytokines, suppressive cytotoxic CD8 lymphocytes, downregulation of antigen processing machinery, the generation of specific inhibitory lymphocytes and the expression of immune checkpoint ligands and/or their receptors also contribute to immune evasion (12, 13). Tolerance to cytotoxic T-cells and upregulation of inhibitory checkpoint receptors can inhibit normal T-cell activation inside the TME allowing the tumor to grow (14).

The intricate mechanism involved in the immunity of head and neck cancers has demonstrated different dynamics depending upon the level of tumor infiltration, tumor mutational burden, tumor stroma, TME and the HPV status of the disease (15–17). A better understanding of the factors associated with an immune suppression in HNSCCs is critical for the development of new therapies or improvements of currently available check point inhibitors such as anti-programmed cell death protein 1/programmed cell death protein ligand 1PD-1/PDL-1 and anti- cytotoxic T-lymphocyte-associated protein 4 (CTLA-4), as well as the selection of candidate patients for immunotherapy. The role of T-cells in HNSCC immunity and the main mechanisms associated with immune evasion, predictors factors related to outcomes and their impact in the tumor response to immunotherapy are discussed below as well as some promising approaches for therapeutical schemes.

## T-cells and the tumor microenvironment in HNSCC

TME is a complex entity composed by the extracellular matrix (ECM), blood vessels and a sort of cells including immune cells, cancer-associated fibroblasts (CAFs), tumor cells and cytokines. There is an interplay between tumor cells and the TME that leads to severe immunosuppression and the proliferation of the malignant tumor (18). Immunosuppressive subsets have been found in HNSCC including tumor-associated macrophages, myeloid derived suppressor cells, and regulatory B and T-cells (10). CAFs produce growth factors like epidermal and vascular endothelial growth factors as well as matrix metalloproteinases, which enable tumor development and progression due to cellular proliferation, invasion, and metastasis. Besides, CAFs promote an immune suppressive TME through the induction of trans-differentiation or polarization of immune cells such as tumor-associated macrophages (TAMs) to pro-tumoral phenotype, as well as by suppressing T-cell infiltration in HNSCC, through secretion and activation of transforming growth factor–β (TGF-β), modulating multiple immune cells leading to a more suppressive phenotype (9, 19).

**Figure 1:**
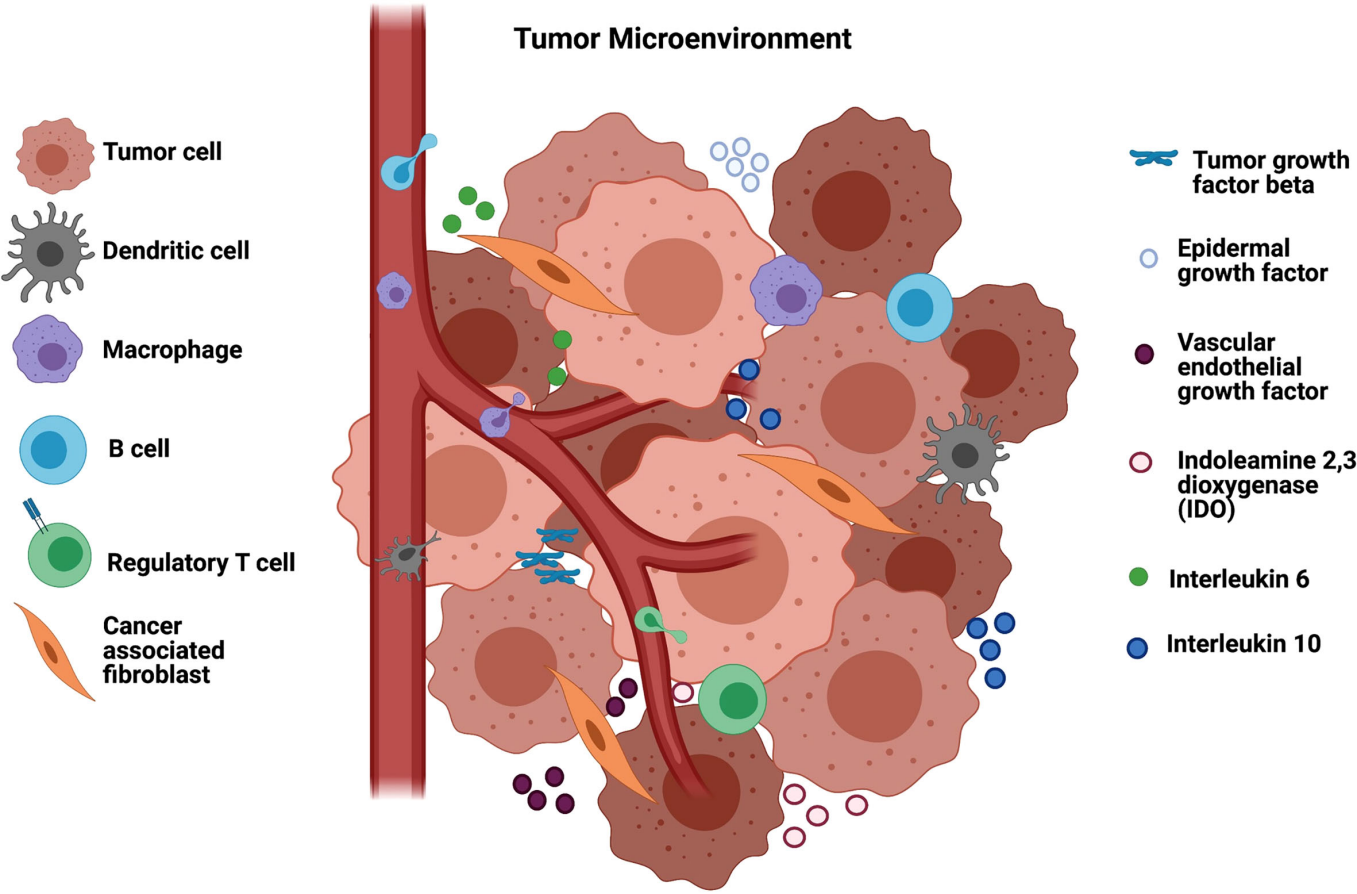
Tumor microenvironment in HNSCC and the immunosuppression. The tumor microenvironment is composed of vessels, B regulatory cells, T regulatory cells and cancer associated fibroblasts (CAF) that modulate macrophages to TAMs and suppress the infiltration of T lymphocytes into the TME. CAFs secrete tumor growth factor beta (TGF-β) that suppress different immune cells as well as epidermal and endothelial growth factors that enable tumor development. Other soluble factors in the TME that mediate immunosuppression include indoleamine 2,3 dioxygenase (IDO) responsible for tryptophan depletion, interleukin 6 (I-L6) and interleukin 10 (IL-10). Figure generated using Biorender (https://biorender.com).

Several other immunosuppressive molecules including indoleamine 2,3-dioxygenase (IDO), which is an enzyme responsible for tryptophan depletion, and some cytokines such as interleukin 6 (IL-6), interleukin 10 (IL-10), and prostaglandin E2, released from tumor or stromal cells, also contribute to modulating immune cell phenotypes in the TME (**Figure 1**). They inhibit T-cell activation and promote immune tolerance, resulting in suppression of anti-tumor immunity (20). Moreover, head and neck tumors cells secrete exosomes containing COX2, TGF-β, PD-1 and CTLA-4 (21) and those inhibitory molecules cause CD8 T cell apoptosis, inhibit CD4 T cell proliferation and increase the frequency of regulatory T-cell (Tregs), compromising the antitumor responses of HNSCC, which accounts for functional defects or apoptosis of T-cells, both circulating and tumor-infiltrating lymphocytes (22, 23).

## Regulatory T-cells in HNSCC

Regulatory Tregs are a subpopulation of CD4 T cells known to suppress the immune response to avoid excessive inflammation and autoimmune disease. Tregs are characterized by the high expression of the interleukin 2 receptor alpha chain, the transcription factor Forkheadbox P3 (FOXP3) and inhibitory molecules such as CTLA-4 and secretion of the anti-inflammatory cytokine IL-10. In cancer, Tregs are known as suppressors of the anti-tumor response, leading to tumor escape (24).

In most cancers, including hepatocellular, renal, melanoma and breast cancer, the high frequency of Treg cells is associated with reduced survival (25). However, in HNSCC the association between the high number of Tregs and disease prognosis is not clear yet. Some studies claim that high frequency of Tregs is related to negative prognostic in HNSCC, whilst others report that high numbers of Tregs are associated with better survival (26, 27). Difference in the frequency of Tregs is observed in HPV+ HNSCC compared to HPV-, with the HPV+ HNSCC presenting higher frequency of Tregs and improved survival. Therefore, distinct factors are related to the numbers of Tregs in HNSCC including tissue modulation (28).

Patients with HNSCC present increased frequency of Tregs in tumor infiltrating lymphocytes (TILs) compared to peripheral blood lymphocytes (PBLs) (29). Similar findings regarding the frequency of Tregs in HNSCC have been described in other studies and they also reported that the numbers of Tregs varies inside the tumor (30). Oropharynx-tonsillar region showed higher frequency of Tregs, followed by base of the tongue, hypopharynx, larynx and other locations (30). The enrichment of Tregs in the tumor seems to be driven by the expression of the chemokine receptors CCR4 and CCR7 that drives their migration and also the chemo attractants such as monocyte chemotactic protein-1 (MCP-1) also known as CCL2 and C-C chemokine ligand 22 (CCL22) (28). CCR7 is highly expressed in TILs but it is also elevated in PBLs of HNSCC patients and in different T cell populations. On the other hand, CCR4 and MCP1 roles in Treg migration to the tumor have been shown through blocking CCR4/MCP1 in mice, which led to reduced frequency of infiltrating Tregs and inhibition of tumor growth (31).

In HNSCC, the frequency of Tregs expressing the inhibitory molecules T-cell immunoglobulin mucin-3 (TIM-3), PD-1 and CTLA-4 are higher in TILs compared to PBLs (29). PD-1 interacts with PD-L1, triggering inhibitory signaling pathways. CD39 and CD73 generate adenosine, which is a suppressive factor. TIM-3 interacts with Galectin-9 and the adhesion molecule carcinoembryonic antigen-related cell adhesion molecule 1 (CEACAM1) and exert its inhibitory function causing cell anergy and phosphorylation of an inhibitory domain on downstream of the T-cell receptor (TCR), resulting in suppression of the TCR signaling (32). Moreover, several molecules related to relevant inhibitory cellular functions have been reported in TILs Tregs. CTLA-4 and CD39 are co-expressed in most TILs Tregs, suggesting that those cells have a higher suppressive function compared to peripheral Tregs in cancer patients (29). β-galactoside binding protein (βGBP) is another molecule highly expressed in Tregs found in oral squamous cell carcinoma. Its blockade reduce their inhibitory function (28). β-GBP interacts with glycoproteins on the surface of T cells blocking their growth and inducing apoptosis. The β-GBP promotes secretion of IL-10 and IL-35 impairing T cell effector function and promoting proliferation of cancer cells. Inhibition of βGBP reduces the levels of IL-10 and IL-35 and impairs cancer cell growth. The evaluation of oral squamous cell carcinoma also shows increased levels of the cytokines IL-10 and TGF-β secreted by Tregs (28). Another study showed that stromal IL-33 regulates the suppressive function of Tregs by inducing the secretion of IL-10 and TGF-β, followed by reduction in the proliferation of effector T cells in patients with laryngeal squamous cell cancer (33).

Since the frequency and function of Tregs seems to be related to HNSCC prognosis, one interesting question is the effect of therapy in Tregs. After chemoradiotherapy the frequency of Tregs remained high and these cells increased their expression of Latency Associated Peptides (LAP), Glycoprotein A Repetitions Predominant (GARP) and CD39 molecules (34), phenotype related to increased inhibitory function. Moreover, Cetuximab therapy increased the frequency of intratumoral Tregs expressing CTLA-4, CD39 and TGF-β, which is correlated with poor clinical outcome. The therapy with Cetuximab also caused the expansion of CTLA-4+ Tregs *in vitro* (29).

## HPV and immune response in HNSCC

HPV strains HPV-16 and HPV-18 have extensively been studied because of their well-known carcinogenic potentials. They are associated with cervical and anal cancers; however, only the HPV-16 strain appears in the etiology of oropharyngeal squamous cell carcinoma. HPV positive head and neck cancers are good targets for cancer immunotherapy due to their intrinsic immunogenicity (35). The role of viral protein expression within HPV+ tumors, as a trigger agent for immune activation and its effect in the immunotherapy response have been discussed elsewhere (36).

The presence of specific immune responses attributed to HPV+ proteins have been associated with better outcome after treatment of HPV+ patients. It has been suggested that as HPV promotes several mutations, that may lead to a more efficient tumor antigens recognition by immune cells. It was already observed that HPV-16-specific CD41 and CD81 T-cells are frequently found in peripheral blood samples from patients with HPV+ HNSCC compared to HPV- HNSCC or healthy controls. Likewise, a different pool of T-cells, including CD41 T-helper Type 1 and 2 cells, CD41 regulatory T-cells and CD81 T-cells, reactive to several HPV-16 E6 and E7 epitopes were observed and the local presence of HPV-16-specific T-cell immunity acts in the antitumor response and support the development of immunotherapy for HNSCC (37, 38).

The role of HPV status regarding a favorable outcome for HNSCC patients remains controversial. Lou et al. have shown that HPV-16 E7 induces the stimulator of interferon genes (STING) degradation *via* an autophagy-dependent mechanism and evasion from anti-tumor immunity through NLRX1-mediated degradation of STING leading to a poor clinical outcomes in patients (39).On the other hand, Nelson et al. claim that although HPV plays a key role as an oncogenic driver of specific patterns of head and neck cancers. It also works as a immunomodulator that impact on the ability of the immune system to identify and target residual cancer cells (38).

## T cell exhaustion and memory formation on HNSCC

**Figure 2:**
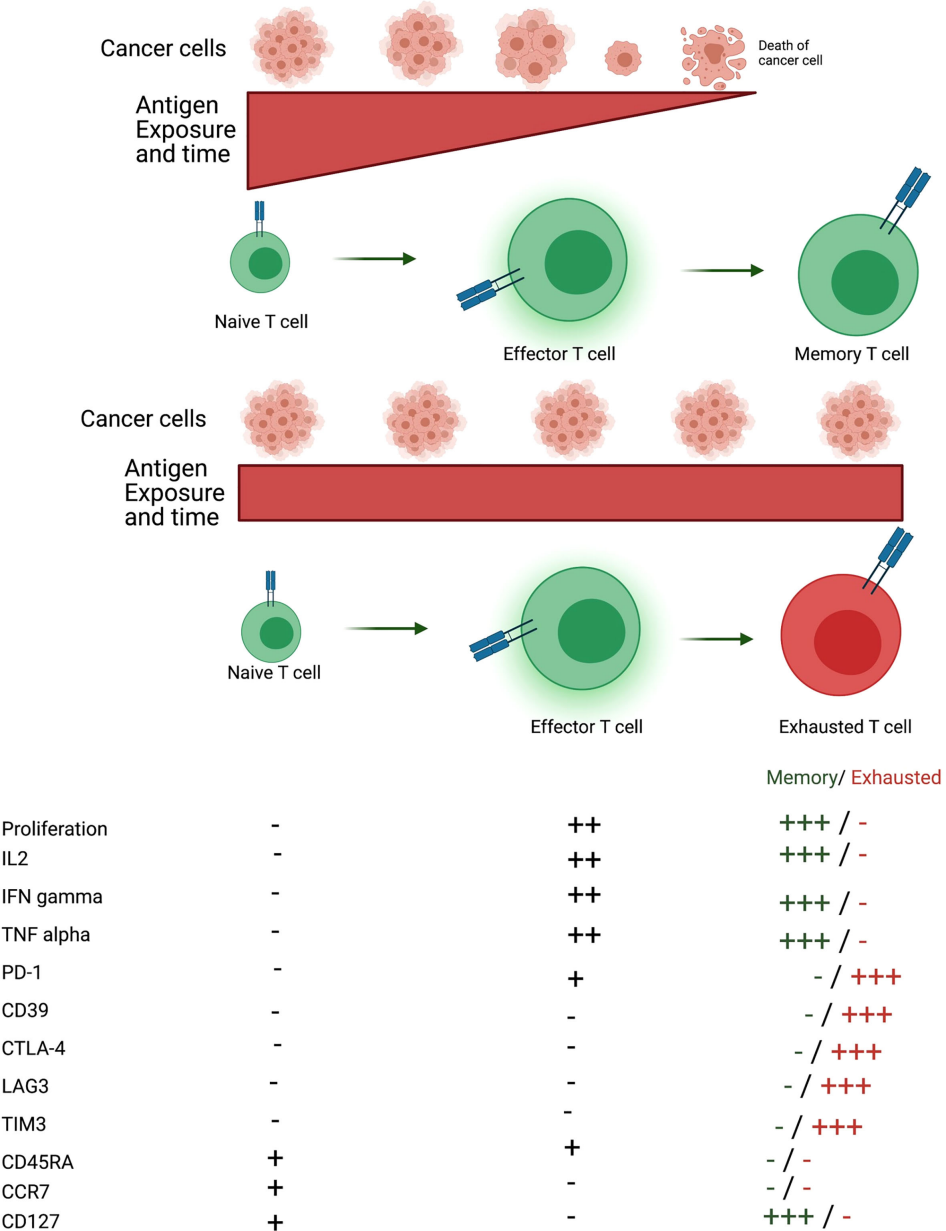
Formation of memory and exhausted T cells. After antigen encounter naïve T cells differentiate into effector T cells that are proliferative and exert their function through secretion of several cytokines such as IL-2, IFN-gamma and TNF-alpha. If the antigen is eliminated, T cells differentiate into memory T cells, which can proliferate and secrete cytokines quickly when reencountering the antigen. On the other hand, if the antigen persists effector T cells differentiate into a state called T cell exhaustion. Exhausted T cells are non-proliferative, not functional and express several inhibitory molecules such as PD-1, CD39, CTLA-4, LAG3 and TIM3. Memory T cells do not express CD45RA and CCR7. There are distinct memory T cell populations, such as effector memory and central memory but effector memory is the population described. Figure generated using Biorender (https://biorender.com).

After antigen exposure, naive T cells differentiate into effector T cells, which are responsible for fighting infections or cancer cells. Effector T cells are very proliferative and functional. Antigen clearance leads to contraction of T cell population and the remaining cells are memory T cells. Memory T cells present increased proliferative response and rapid effector function in case of secondary response to previously eliminated antigen (40). However, a prolonged antigen exposure, such as chronic infections and cancer, leads T cells to an unresponsive state called T cell exhaustion. Exhausted T cells are not functional, express inhibitory molecules and present reduced proliferative capacity (**Figure 2**) (41).

Exhausted T cells are characterized by the expression of inhibitory molecules, such as PD-1, CTLA-4, T cell immunoglobulin and ITIM domain (TIGIT), Lymphocyte activation gene 3 (LAG3), TIM-3 and others, that negatively regulate their response, as well as reduced secretion of cytokines and cytolytic molecules (42). Several studies described T cell exhaustion in distinct types of cancer, including liver, lung and head and neck (43–45). However, it is not known at which stage the cells acquire this unresponsive state.

The expression of distinct inhibitory molecules by exhausted T cells varies when comparing T cells from the peripheral blood and tumor infiltrated lymphocytes. TIGIT interacts with CD155 leading to T cell suppression. Both CD4 and CD8 T cells from periphery and also TME express the high levels of TIGIT with higher levels being expressed by TILs (46). TIM-3 is another inhibitory molecule expressed by exhausted T cells and is highly expressed on tissue samples of HNSCC patients. The expression of TIM-3 has been linked to metastasis but it has not been linked to patient survival. On the other hand, the blockage of TIM-3 expression using TIM-3 monoclonal antibody leads to inhibition of tumor growth in HNSCC mice model (47). The inhibitory molecules CD73 converts adenosine monophosphate (AMP) into adenosine, which binds to G coupled receptors and causes tumor growth, increased cell migration and invasion. Gene expression analysis on human HNSCC samples shows high expression of CD73 in tumor infiltrating immune cells, which correlates with poor prognosis of those patients (48). The inhibitory molecule LAG3 was also found to be highly expressed on CD4 and CD8 TILs on samples of HNSCC patients and their expression correlate with poor outcome (49). HNSCC tissues express the high levels of the PD-1 ligand, PD-L1. The interaction PD-1/PDL1 is one of the factors responsible for the formation of HPV+ HNSCC better outcome after its inhibition. In fact, tumor infiltrated CD8 T cells express higher levels of PD-1 in HPV+ HNSCC than in HPV- HNSCC (50). The frequency of T cells expressing PD-1 is higher in the tumor tissue compared to peripheral blood of HNSCC patients as well as the healthy individuals (44).

HPV is responsible for the expression of PD-L1, leading to immune evasion and HPV persistence (51). CD8 T cells in HPV+ HNSCC express higher levels of genes associated with T cell exhaustion, such as CD39, LAG3, PD-1, TIGIT and TIM3 compared to HPV- HNSCC. The expression of at least one of those inhibitory genes is related to better patient survival in HPV+ HNSCC but that correlation was not observed in HPV- HNSCC. One explanation is that expression of checkpoint molecules indicates tumor antigen specificity. Higher levels of co-expression of at least two of those molecules were also observed in CD8 T cells of HPV+ HNSCC compared to HPV- HNSCC, which suggests T cell anti-tumor immunity and contribution to long term remission (52). CD8 T cell exhaustion in HPV+ HNSCC has been linked to the expression of the HPV-16 antigen (53).

The dichotomy between T cell exhaustion and memory T cells as well as the relevance of memory formation to therapy development highlight the importance of evaluating memory T cells in HNSCC. Comparison between healthy individuals and patients with HNSCC shows that patients with HNSCC present higher frequency of both CD4 and CD8 memory T cells. However, the difference is more pronounced when comparing CD4 T cells. This suggests that CD4 and CD8 T cells differentiate differently in response to HNSCC or the lifespan of CD8 memory T cells is shorter compared to CD4 memory T cells (54).

There are different populations of memory T cells such as effector memory and central memory T cells. Effector memory T cells present a quick effector function response but a shorter life span. On the other hand, central memory T cells do not present effector function, but can quickly differentiate into an effector population and proliferate, while their life span is longer compared to effector memory T cells (55). Patients with HNSCC present higher frequency of effector memory T cells and reduced frequency of naive T cells in the peripheral blood compared to healthy individuals (56). Higher frequency of central memory T cells is also observed in HNSCC patients compared to healthy individuals (57).The evaluation of memory T cell populations in blood samples with HPV infection shows higher frequency of effector memory T cells and lower frequency of naïve T cells in HPV+ HNSCC patients compared to HPV- HNSCC patients. There is no difference in the frequency of central memory T cells considering HPV status in HNSCC (57). Effector memory T cells are also found in high frequency in the TME, but no difference is observed in the frequency of this population comparing HPV+ HNSCC and HPV- HNSCC (44).

## Immune checkpoint inhibitors in HNSCC treatment

TCRs can identify and target cancer cells; however, several tumors possess resistance mechanisms by using checkpoint blockade molecules weakening immune recognition and attack. On the other hand, immunotherapy can reactivate T cells to target tumor cells. Several immunotherapies have been approved for the treatment of head and neck cancer, including immune checkpoint inhibitors for the management of recurrent or metastatic tumors (58, 59).

The main role of immune checkpoint inhibitors is hampering the interaction between inhibitory receptors and their ligands, such as PD-1/PD-L1, CTLA-4/CD80/Cd86, TIM3/Gal9/CEACAM1 and others. PD-1 is a member of the CD28 receptor family which is expressed on activated T- and B-cells, monocytes, and a subset of thymocytes working as an inhibitor of T cell responses. The effectiveness of a given immunotherapy agent depends on the knowledge of the target molecule mechanism of action, which leads to the identification of suitable biomarkers. It is already known that the interaction between PD-1 and its ligand PD-L1 negatively regulates immune responses by decreasing cytokine production and inducing T lymphocyte anergy and apoptosis (42). Strauss et al. suggested that a deletion of PD-1 in myeloid cells during differentiation to effector Antigen-Presenting Cells (APCs) might be a key mechanism by which PD-1 blockade mediates antitumor function by reprogramming T cell responses (43).

Some FDA approved agents are ongoing clinical trials and have demonstrated improvements in patient outcomes for advanced/metastatic HNSCC. Nivolumab and Pembrolizumab are IgG4 anti-PD-1 monoclonal antibodies designed to block co-inhibitory signaling through the PD-1/PD-L1 axis (44). Besides, Pembrolizumab enhances K+ channel activity, Ca^2^+ fluxes and chemotaxis of CD8+ T cells in patients with HNSCC. Improved cytotoxic T cells response (45) and enhanced patient`s overall survival has been observed after Pembrolizumab administration (46). Other IgG1 anti-PD-L1 antibodies Durvalumab, Avelumab and Atezolizumab, designed to reduce antibody-dependent cellular cytotoxicity (ADCC) are currently in clinical trials for HNSCC treatment, and other inhibitory immune checkpoints including TIM-3, IDO, KIR, and TIGIT are under investigation as well (47–49).

**Figure 3:**
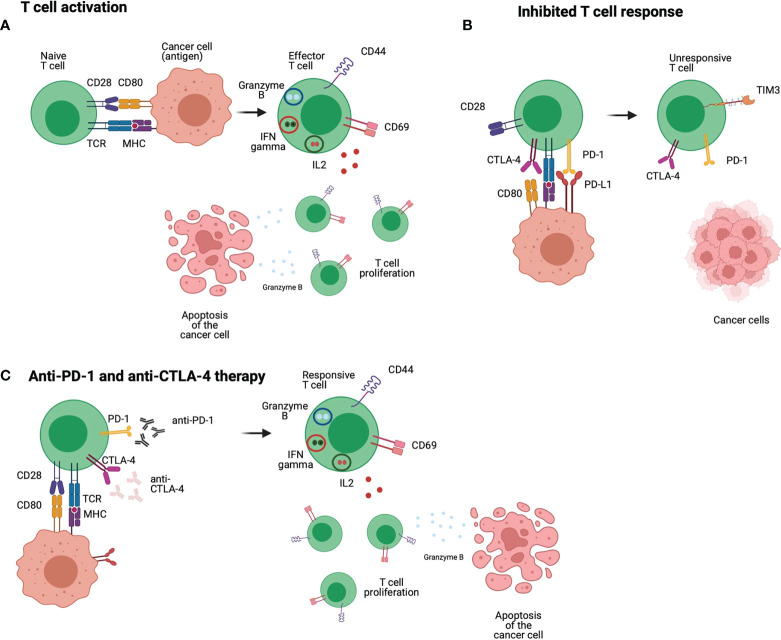
Anti-PD-1 and anti-CTLA-4 immunotherapies: T cells receive different stimulations that lead to an effective response, the first comes from the interaction between TCR and MHC, but co-stimulation is also required such as the interaction of CD28 and CD80/86. The interaction between those molecules lead to positive signal and differentiation of T cells into a proliferative and responsive population **(A)**. In HNSCC patients, T cells express inhibitory molecules such as PD-1 that interacts with PD-L1 leading to a negative signal to the T cells. CTLA-4 is highly expressed in HNSCC T cells, and it interacts with CD80/CD86 blocking co-stimulation signals to the T cell. Therefore, the T cell is non proliferative and unresponsive **(B)**. Anti-PD-1 and anti-CTLA-4 are used to block the inhibitory molecules PD-1 and CTLA-4 and avoid the inhibitory signals received by the T cell. In this case, the T cell receives positive stimulatory and co-stimulatory signals and can exert their effector function to eliminate tumour cells **(C)**. Figure generated using Biorender (https://biorender.com).

CTLA-4 is expressed on the activated T cells surface, binding to B7 protein, cell-surface protein that regulate immune responses, to avoid the interaction with the co-stimulatory CD28. It is an essential component of antigen-specific naïve T cell co-stimulation during initial priming by DCs, that leads to a negative regulation of T cell proliferation and IL-2 production. For this checkpoint inhibitor, anti-CTLA-4 Ipilimumab and Tremelimumab have been investigated, however, the latter is not approved by FDA for the treatment of HNSCC yet (**Figure 3**) (50). It has been reported that CTLA-4 blockade lead to better efficiency with long term remission and increased response in patients (51). CTLA-4 blockade on Tgfbr1/Pten 2cKO HNSCC mouse model showed reduction of the tumor burden of head and neck without additional cytotoxicity. CTLA-4 blockade lead to reduced frequency of regulatory T cells and increased T cell function (52). Randomized phase III trials showed that Ipilimumab present great curative effect in patients with melanoma. In HNSCC patients Ipilimumab reduced the suppression of natural killer cells by regulatory T cells. Tremelimumab has been successfully used to treat patients with melanoma and other cancers (51).The Combination ICB targeting PD-1 and CTLA-4 also can be an interesting strategy. A nonrandomized phase Ib/IIa trial (NCT03003637) investigated the safety, feasibility and efficacy of ipilimumab and nivolumab neoadjuvant to surgery in patients with advanced or recurrent HNSCC. The combination between immunotherapies prior to surgery show to be an effective and safe regimen for patients with resectable and predominantly HPV-negative HNSCC, resulting in a major pathological response (90–100% response) in 35% of patients after treatment (53).

Although immune checkpoint inhibitors have provided an option for cancer treatment to patients with recurrent/metastatic tumors, important immune-related adverse events have been reported such as dermatitis, hypothyroidism, pneumonitis and hepatitis (54, 55). The future of checkpoints immunotherapy seems promising; however, the main therapeutic challenge is to reach the poorly lymphocyte infiltrated tumors (56).

## Gene mutations and Cell signaling in HNSCC

The HNSCC is a malignancy associated with two distinct oncogenic pathways, drive by either exposure to typical carcinogens or infection of HPV. HPV encodes the potent oncoproteins E6 and E7, which bypass many important oncogenic processes and result in cancer development. In contrast, HPV-negative HNSCC is developed through multiple mutations in diverse oncogenic driver genes (57). The cellular responses observed on HNSCC are mediated by biochemical signal transduction and they may reflect genetic mutations. Alterations in the gene sequence implicates in overexpression or under expression of proteins in specific pathways that mediate cell survival, proliferation, and migration (60). The main genetic mutations in HNSCC are on tumor protein p53 – TP53 (71%), fat atypical cadherin - FAT1 (23%), cyclin dependent kinase inhibitor 2A - CDKN2A (22%), Phosphatidylinositol-4,5-Bisphosphate 3-Kinase Catalytic Subunit Alpha - PIK3CA (18%), Notch receptor 1 - NOTCH1 (17%) and HRAS (6%), followed by rare mutations that require further evaluation (61). These play a key role in the RTK/RAS/PI3K pathway, and an accumulative effect of this pathway results in tumorigenic alterations in cellular functions including cell growth, differentiation, survival and migration (62).

TP53 mutations are the most common in HNSCC and they are associated with short survival time and resistance to radiotherapy and chemotherapy (63). Mutations of TP53 leads to its inactivation and interaction with non-mutated TP53 inhibiting its activity (64). TP53 is a transcription factor that positively or negatively regulates the expression of several genes (65). The TP53 is part of a network of genes, called TP53 pathway, which gets activated after intrinsic and extrinsic stress such as DNA damaging caused by gamma and UV radiation, reaction with oxidative free radicals, alkylation of bases, depurination of DNA; hypoxic conditions; and metabolic stress (66). TP53 is activated by phosphorylation and acetylation, which are mediated by protein kinases and histone acetyltransferases (67). Among the proteins that activate TP53 are casein kinase I (CKI), casein kinase II (CK2), ataxia-telangiectasia mutated (ATM), the mitogen activated protein kinase p38 and Jun N-terminal kinases (JNK) (68). Each one of those kinases have been described to phosphorylate TP53 in distinct sites and different stress may induce the activation of a specific kinase that will end up phosphorylating TP53 (69). The acetylation has also been described to activate TP53 (70). After its activation, TP53 regulates the expression of genes such as plasminogen activator inhibitor-1 (*PAI-1*), thrombospondin and maspin together with TP53 interacts with FAS/CD95 mediating its translocation to the plasma membrane resulting in increased cell death (66). TP53 also induces the transcription of BCL2 associated X gene (*BAX*), which is a proapoptotic member of the BCL2 family (71). TP53 regulates cell cycle causing G1 arrest by mediating *p21* gene that inhibits cyclin E-cdk2 (72). Cells are also arrest at G2 phase of cell cycle through the synthesis of 14-3-3 sigma mediated by TP53 (73). 14-3-3 sigma binds to CDC25C and keeps it in the cytoplasm, avoiding it from activating B-CDC2 in the nucleus and blocking the cells in G2 phase (66).

*FAT1* is one of the most frequently mutated genes on HNSCC. Its mutation has been linked to loss of activity, increased cell growth and proliferation. FAT1 is a transmembrane and its intracellular domain interacts with β-catenin, preventing β-catenin nucleus translocation and transcription of target genes (74). The atypical activation of the Wnt/β catenin signaling pathway promotes tumorigenesis and cancer cell proliferation (75). Mutations on *FAT1* results in loss of ability of FAT1 to interact with β-catenin, leading to activation of the Wnt signaling pathway, transcriptional activity of β-catenin and increased expression of wnt genes such as *cyclin D1* and *zinc finger E-box binding homeobox 1* (*Zeb1*) (76). Cyclin D1 regulates cell cycle progression from G phase and the transition between G_1_max/S (77). Zeb1 regulates cancer cell differentiation and metastasis. Moreover, zeb1 has been described as mediator of chemoresistance (78). Therefore, increased activity of β-catenin, increases the expression of cyclin D and Zeb1, leading to metastasis and chemoresistance. FAT1 interacts with Ena/VASP, which are regulators of actin dynamics (79). Therefore, FAT1 regulates the cytoskeleton and mediate cell migration. Mutation and low expression of *FAT1* are predictors of poor prognosis in patients with HNSCC. However, *FAT1* mutation has been linked to better prognosis in HPV- HNSCC patients (80).

The third most common mutation on HNSCC is on *CDKN2A* gene. Approximately 90% of HPV- HNSCC present low expression of *CDKN2A*, which is usually occurring due to mutations, loss of heterozygosity and hyper methylation of the gene (81). *CDKN2A* encodes one of the tumor suppressor proteins called p16^INK4a^, which is a negative regulator of cell cycle (82). p16^INK4a^ binds to CDK4 or CDK6, leading to a conformation change and inhibiting the interaction between CDK4 or CDK6 and cyclin D (83). The inhibition of the complex CDK4/CDK6 and cyclin D maintains retinoblastoma protein (Rb) hypo-phosphorylated and bound to E2Fs (84). The complex Rb/E2Fs repress the cell cycle and arrest the cells in G1 (84). CDKN2A also encodes p19^ARF^, which interacts with p53 (85, 86). P53 levels increase due to DNA damage, nucleotide deprivation and hypoxia (87). Then, nuclear p53 levels are elevated, stabilized and activated, leading to cell cycle arrest or apoptosis (88). The protein H2DM is responsible for capturing p53 from the nucleus to the cytoplasm, where it is degraded (89). P14^ARF^ sequester H2DM to the nucleus and inhibits its interaction with p53 (90). Therefore, p14^ARF^ prevent cell growth not only by inhibiting H2DM but also by stabilizing p53 (91). The activation of p14^ARF^ is dependent of E2Fs, which links it to Rb and the cell cycle regulation as previously described (92).

Another common mutation on HNSCC is found on *PI3KCA*, which is equally presented in HPV- and HPV+ HNSCC (93). *PI3KCA* encodes the catalytic subunit of phosphatidylinositol 3 kinase (PI3K), called p110 alpha (94). PI3K is a member of the PI3K signaling pathway, which is activated by stimulation of tyrosine kinase receptors such as ErbB, G protein-couple receptors and EGFR (95). Activation of PI3K triggers the catalytic function of p110 alpha, causing the phosphorylation of phosphatidylinositol 4,5 biphosphate (PIP2) to phosphatidylinositol 3,4,5 triphosphate (PIP3) (96). PIP3 triggers the activation of AKT dependent and independent signaling pathways. Activated AKT regulates cell proliferation, growth, survival and metabolism (97). PI3KCA mutation leads to increased PI3K activity and deregulation of this pathway is related to metastasis and HNSCC poor prognosis (93, 94). Therefore, several PI3K inhibitors are under evaluation to be used as monotherapy or in combination with radiotherapy and/or chemotherapy (93). Some inhibitors used as monotherapy have shown reduced patient response, whilst other inhibitors when combined with other therapies were successful (98, 99). Targeting the PI3K signaling pathway is a promising strategy and distinct approaches must be evaluated.

Deregulated NOTCH pathway activity is also observed in HNSCC due to mutation on *NOTCH1*. NOTCH1 regulates cell differentiation, proliferation, and apoptosis (100). NOTCH1 activation starts with the interaction between EGFR and ligands (Jagged 1,2 (JAG1, JAG2), delta-like ligand 1,3,4 (DLL1, DLL3, DLL4)) expressed by neighbor cells (101). This interaction triggers conformational changes on NOTCH1 allowing it to be cleaved by a disintegrin and metalloproteinase (ADAM) and γ-secretase complex (102). After cleavage, NOTCH1 intracellular domain migrates to the nucleus where it regulates gene transcription (103). The main targets of the NOTCH signaling pathway are basic-helix-loop factors Hey and Hes families (104). Most *NOTCH1* mutations leads to its inactivation and suggests that it has a tumor suppressor function (105). At the same time NOTCH genes are upregulated in HNSCC compared to healthy tissues (106). Therefore, NOTCH1 has a bimodal function as oncogene and tumor suppressor (107). *NOTCH1* mutations in HNSCC have been associated to worse prognosis, overall survival and disease-free survival compared to wild type (106). Increased *NOTCH1* expression has also been associated to HNSCC progression (106). However, no correlation has been observed between mutated *NOTCH1* and wild type comparing recurrence and invasion (107). *NOTCH1* mutation has also been associated to higher sensitivity to radio and chemotherapy (101). Nevertheless, therapies targeting mutated *NOTCH1* in HNSCC is challenging since most mutations do not lead to NOTCH1 increased activation (103). Further studies and different strategies to target NOTCH in HNSCC need to be developed to provide better therapeutic options.

Mutations on *HRAS* are also observed in HNSCC but they are less frequent (108, 109). HRAS is a member of the GDP/GTP binding proteins. RAS is a member of the mitogen activated protein kinase (MAPK) signaling pathway and presents different isoforms such as HRAS, KRAS and NRAS (110). Likewise, distinct receptors trigger the activation of the MAPK signaling pathway including EGFR (111), and receptor stimulation leads to HRAS binding to GTP, which causes its activation followed by interaction with RAF (112, 113). The complex RAS-RAF induces the phosphorylation of other kinases including mitogen activated protein kinase kinases (MEK) and those phosphorylate other kinases including extracellular signal-related kinases (ERKs) (114). The mutations observed on HRAS in HNSCC are activating, keeping it in GTP state through inhibiting its GTPase activity (115). Thus, activation state of HRAS in HNSCC indicates an ideal scenario for the use of inhibitors. In the past inhibitors of the MAPK pathway showed reduced cell growth on *in vitro* studies but failed in efficacy and caused toxicity in clinical trials (116). The reasons behind the lack of efficiency could be caused by drug resistance, drug potency issues and wrong mechanism of inhibiting MAPK signaling pathway (117). Interference on the MAPK signaling using inhibitors is known to lead to feedback and signaling cross talk, which makes the reactivation of the pathway (117). In recent years, new inhibitors have been developed presenting greater potency and not causing feedback, including MEK and RAS inhibitors (116). Some of those inhibitors are still on clinical trial studies but they are showing promising results (116). Targeting the MAPK signaling pathway in HNSCC patients is a relevant strategy, but identification of the ideal target and the precision of the inhibitor are necessary for therapy efficacy. Since those inhibitors are not specific, the effect of inhibiting the MAPK signaling on healthy cells should also be considered.

The molecular differences between HPV-positive and HPV-negative HNSCCs are substantial and can lead to differential immune responses. HPV-positive express viral proteins as foreign antigens, in addition to other neoantigens created by viral integration and induced mutagenesis. In contrast, HPV-negatives lacks foreign antigens, instead they are generated from extensive random mutations or overexpressed cellular genes (57). Comprehensive genetic alterations profiling leads to the development of “personalized” or “precision” medicine and can promote targeted therapies due to number of different pathways are altered in HNSCC (118).

Immunotherapy has emerged as a promising therapeutic avenue in HPV-positive HNSCC due to chronic viral infection resulting in a unique, non-self, antigenic target (119). Nevertheless, recent immunotherapy trials have not found any clear benefits of using immune checkpoint inhibitors to treat HPV-positive patients compared to HPV-negative patients (57).The biological rationale for antitumor immunotherapy specifically in HNSCC is built upon several observations. The HNSCC has a relatively high tumor mutation burden, the TME is generally immunosuppressive and frequently infiltrated with immune cells that could be targeted towards anti-tumor effects and the HPV-positive HNSCC provides a convenient therapeutic and antigenic target (120).

## CAR-T cells as immunotherapy for HNSCC

The standard therapies to treat patients with HNSCC consist mainly of chemotherapy and radiation therapy, and several HNSCC have demonstrated resistant to these treatments, which are responsible for poor survival rates and tumor recurrence (121, 122) Although both therapies are combined to maximize tumor control, it only increases overall survival by 5% (123). Therefore, new therapies are needed to treat HNSCC to achieve better efficiency, less toxicity and better quality of life.

**Figure 4:**
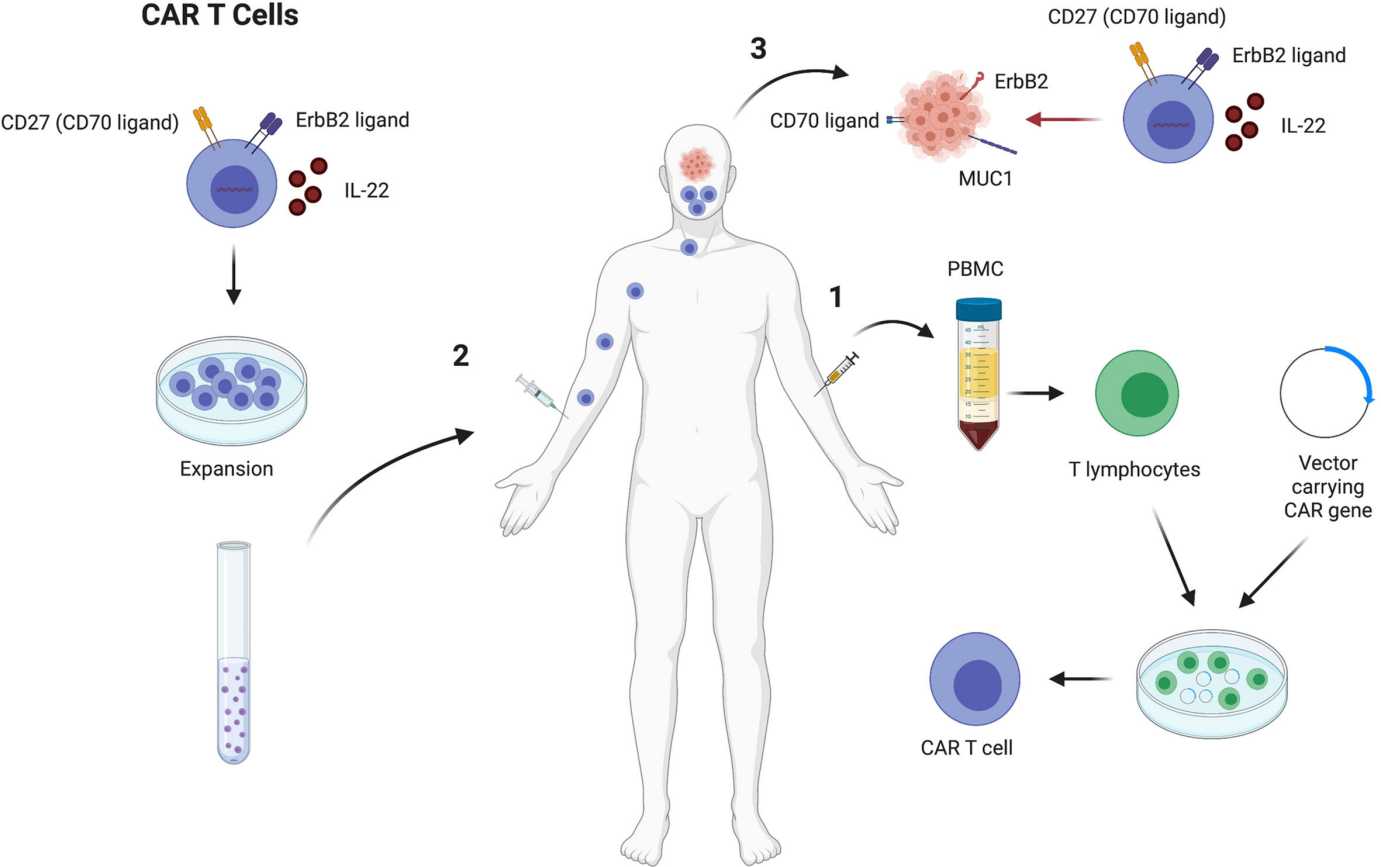
CAR T cell therapy to treat HNSCC that express high levels of CD27, ErbB2 and secrete IL22. CAR T cells generation starts by drawing blood from HNSCC patients, followed by PBMC separation. Thereafter, T cells are put in culture in the presence of a vector to induce the cells to express molecules that can target the tumor cells, such as CD27, ErbB2 and IL22 (1). Chimeric antigen receptor T cells are expanded *in vitro* and injected back in the HNSCC patient that previously provided the T cells (2). The CAR T cells will target the tumor cells through secretion of IL22 that increases the MUC1 expression on the tumors and increase T cell function, and the interactions between CD27/CD70 and ErbB2/ErbB2 ligand, which drives the T cells to the tumor environment (3). Figure generated using Biorender (https://biorender.com).

Chimeric antigen receptor (CAR) T cell technology have recently transformed the cancer immunotherapy field. CAR T cells recognize specific antigens on the surface of the tumors and eradicate them. The strategy starts with identifying specific makers on tumors that can be used as a target. Thereafter, T cells are isolated from patient’s sample and genetically altered using viral vectors to express specific receptors on their surface called chimeric antigen receptors (CARs). Genetical alterations on the T cell will depend on the molecule to be targeted in the tumor. CAR T cells can be expanded *in vitro* and injected back into the patient to recognize and target tumor cells (**Figure 4**) (124).

Several studies have demonstrated that specific molecules expressed by HNSCC can be targeted. Among the markers described to be potential targets of CAR T cells in HNSCC are CD276, EGFR, MICA, MICB, MAGE-A4, FAP, EPCAM, CD70, B4GALNT1 (125). CD70 is differentially expressed in distinct tumor subtypes and in individual tumors as well. CD70 is highly expressed in HNSCC tumor biopsies (20%) and 75% of specimens showed high CD70 expression on tumor surface. When CAR T cells were generated to target cancer cells that express CD70, HNSCC expressing CD70 were efficiently killed and CD70 negative tumor cells were not targeted (125). Another study showed that MUC1 is highly expressed in cancer tissues compared to adjacent non neoplastic tissue. In this case, CAR T cells that secret IL-22 play a relevant role by increasing the expression of MUC1 on HNSCC and increasing T cell function (126). Another strategy involves generating CAR T cells that express HER2 receptors to drive T cell responses towards the cancer cells. HER2 is also known as ErbB2, which is a receptor tyrosine kinase expressed in most epithelial cell layers and mediate cell differentiation. ErbB2 is overexpressed in several human cancers and is a potential target for CAR T cells. Evaluation of HNSCC shows that CAR T cells targeting ErbB2 results in 56% decreased tumor size (**Figure 4**) (127).

A clinical study in phase I of T4 CAR T cells to treat patients with HNSCC is ongoing. The T4 immunotherapy consists of generating T cells that express T1E28 ζ composed of ErbB ligand linked to CD28 and CD3 endodomain; and 4αβ, which is an IL4 chimeric receptor. T1E28 ζ provides broad anti-tumor range and reduced possibility of antigen escape. 4αβ allows the expansion and enrichment of the CAR T cells during production phase. Preliminary data has shown that intratumoral injection of T4 CAR T cells is safe with no toxicity. T4 immunotherapy led to 69% disease control even with rapidly progressing tumors and in patients with advanced HNSCC (128).

## Immunotherapy based on nanotechnology

Despite advances in the development of immunotherapies against cancer, some limitations are also associated and block progress in clinical studies. Among the limitations, the induction of destructive autoimmunity is one of the main challenges to overcome, since the treatment can generate autoimmune events with damage to several organs (129, 130). The nonspecific interaction of immunostimulating agents with proteases, nucleases, and immune cells not only reduce immunostimulating capacity, but also can result in safety concerns and lead to excessive inflammation, toxicity, and hypersensitivity (131). Furthermore, the ineffective delivery of immunostimulating agents to immune cells can reduce their action. Immunostimulating agents often suffer from sub-optimal pharmacokinetics, vulnerability to biodegradation, and impaired cell targeting when administered directly into the body (132).

In this context, nanotechnology emerges as a promising field for overcoming problems related to traditional immunotherapy against cancer and, thus, increasing its effectiveness. In general, immunotherapeutic agents are complexed with biocompatible nanomaterials. This kind of formulation can extend the half-lives of anti-tumor agents, preventing biodegradation and increasing their biological stability; improve immune tolerance through the reduction of nonspecific cellular interactions; and enhance immune stimulation in targeted delivery to specific immune cells, since most immunostimulating agents are only relevant to certain subsets of cells (133). Moreover, due to the small size, nanoparticles can passively accumulate in high concentrations into solid tumors through the enhanced permeability and retention (EPR) effect exhibited by tumor cells (134, 135).

To be used with nanocarriers of immunotherapeutic agents, organic and inorganic nanoparticles have specific sizes, shapes and surface characteristics, which directly influence the delivery of therapeutic agents (136). In particular, polymeric nanoparticles, liposomes, metallic nanoparticles and carbon nanotubes stand out as carriers for immunotherapy, which can improve the antigen presentation process and lead to better T cell stimulation (133, 137).

Regarding the treatment of HNSCC, the combination of nanoparticles with checkpoint blockade cancer immunotherapy seems a promising direction for a less toxic, tailored cancer treatment and to improve delivery of cancer antigens and adjuvants (138). There is a growing tendency towards the application of nanotechnology to improve immune checkpoint inhibitors effects. For instance, gold nanoparticles are of particular interest due to their remarkable optical properties and neglectable associated cytotoxicity. One of their main properties is the ability to increase volume/area surface ratio, allowing immunotherapeutic antibodies binding to gold nanoparticles at very low concentrations (139, 140).

Cetuximab is an anti-EGFR monoclonal antibody that has been used in recurrent/metastatic HNSCC treatment. Previous studies have shown that cetuximab-coated HNC cells induce NK cells, promote DC cross-talk and expand EGFR-specific cytotoxic T cells (141). Although cetuximab has demonstrated to increase overall survival rate, treatment-related toxicity is a common clinical event (129). It was already demonstrated that stable gold nanoparticles coated with 200 µg of cetuximab target EGFR and lead to apoptosis in human squamous cell carcinoma, suggesting their application as a possible agent to overcome immunotherapy-related side effects (142). Albumin is another versatile and stable biomaterial for nanoparticle synthesis and tumor therapy, as albumin can specifically bind to receptors overexpressed in cancer cells, such as gp60 (60 kDa glycoprotein receptor) and SPARC (secreted protein, acidic and rich in cysteine) and actively increase the internalization of nanoparticles (143). Study demonstrated that Nanobody-albumin nanoparticles (NANAPs) coated with bifunctional polyethylene glycol 3500 (PEG) and functionalized with anti-EGFR nanobody (EGa1) to delivery of a multikinase inhibitor17864-Lx- a platinum-bound sunitinib analogue- are able to increase binding 40-fold in EGFR positive HNSCC -14C cells (14C) compared to nanoparticles without EGa1 (144). Sunitinib has immune modulating properties, which include increasing the influx of lymphocytes and DCs into the tumor, while decreasing intratumoral frequencies of Tregs and myeloid-derived suppressor cells (145). Intracellular targeting of EGa1-PEG nanoparticles loaded with 17864-Lx leads to successful release of the kinase inhibitor into the cell and inhibition of cell proliferation, whereas untargeted formulations had no antiproliferative effects on 14C cells. These results demonstrated that nanoparticles were effective for the delivery of T cell therapeutic agents for the treatment of EGFR-positive cancers (144).

Nanoparticles can also act as potent immunostimulators and at the same time as an intelligent carrier for effective delivery of immune checkpoint inhibitors (146). The administration of tandem peptide nanocomplex (TPNC) carrying CpG DNA ligand of TLR9s (iTPNC) can suppress tumor growth in several animal models of various cancers, resulting in an abscopal effect on distant tumors, and improving responsiveness to anti-CTLA-4 treatment. In this study, it was shown that the enhancement of the effect of CTLA-4 is mediated largely by macrophages. However, TLR9 is expressed by several other immune cells, including certain subsets of DCs and T cells, so it is possible that the effects of iTPNC treatment may be mediated through the involvement of TLRs in T cells (147). In addition, the nanocomplex formulation allowed for dramatic reductions in the dose required to produce the therapeutic result, which minimizes the risk of off-target immune activation and various other side effects associated with systemic inflammatory signaling (147). IL-1α-loaded polyanhydride nanoparticles proved to be a safe and novel immunotherapeutic strategy as a single agent and for use in combination with cetuximab for HNSCC therapy. Based on the anti-tumor properties of IL-1 ligands, recombinant IL-1 ligands were previously utilized as anti-cancer agents (148). However, dose-related side effects such as hypotension, fever, vomiting and abdominal pain although manageable are reported (148). IL-1α-loaded polyanhydride nanoparticles did not affect the anti-tumor efficacy of cetuximab and their combination with cetuximab induced a T cell-dependent anti-tumor immune response and may represent a novel immunotherapeutic strategy for EGFR-positive HNSCCs (149). This study observed increased levels of CD8+ T cells and decreased PD-1 + CD4+ and CD25 + CD4+ T cells in spleens of BALB/c mice administered cetuximab + IL-1α-loaded polyanhydride nanoparticles compared to control. Furthermore, depletion of CD4+ and CD8+ T cells significantly reversed the effect of cetuximab+IL-1α-NP suggesting that IL-1α in combination with cetuximab can induce a T cell-dependent anti-tumor immune response (149). The recurrence of HNSCC after surgical resection continues to be a challenge to cancer treatment (150). The photothermal therapy (PTT) can increase the infiltration of immune cells to make tumors more susceptible to cancer immunotherapy (151). Nanocomposite comprised of oxidized bacterial cellulose, thrombin, and gold nanocages (AuNCs) containing PD-1 antibody (TB/αPD-1@AuNCs/OBC) was constructed to be used as a versatile implant for avoiding the recurrence of HNSCC after resection. The therapeutic system could induce tumor pyroptosis and enhance antitumor immune response by increasing T-cell infiltration and reducing the immune suppressive cells (152). The direct delivery of immunomodulatory agents into T cells to control the immunosuppressive TME in melanoma was carried out applying cationic lipid-assisted PEG–PLA-based nanoparticles delivering siRNA into T cells *in vitro.* The results demonstrated reduced CTLA-4 mRNA and protein levels and activation of T cells as well as increased percentage of effector CD4+ T cells and CD8+ T cells and decreased ratio of CD4+ FOXP3+ Tregs inhibiting tumor growth and prolonged survival time in mice with melanoma (153). Besides, Chen et al, observed that tumor-associated Tregs can be preferentially depleted *via* iron-oxide nanoparticles combining anti-CTLA-4 immunotherapy with photodynamic therapy (154). Hafnium oxide nanoparticles (NBTXR3) activated by radiotherapy increase radiation dose deposit within cancer cells compared to radiotherapy alone. NBTXR3+RT demonstrated an immunogenic cell death-mediated abscopal effect with immune cell infiltration in some tumors treated. NBTXR3 is currently being evaluated in 7 clinical trials, including a phase I/II study in elderly frail patients with locally advanced HNSCC in combination with anti-PD-1 therapy (155).

Drug delivery is a well-known approach for antineoplastic antigen and anti-tumor agents to cancer cells through nanomaterials. For instance, bacterial toxins are among the bacterial components with strong antitumor activity. Liposome-Encapsulated CpG (cytosine-phosphorothioate-guanine) Oligodeoxynucleotides (CpG-ODNs) exhibit potent immunostimulating activity by binding with Toll-like receptor 9 (TLR9) expressed on DC cells and activating NK cells, NKT cells and enhance expression of the early activation molecule CD69 on conventional T cells (156). Iron oxide nanoparticles also can simultaneously promote the reprogramming of tumor-associated macrophages to a pro-inflammatory profile and effectively deliver the ovalbumin antigen (OVA) to dendritic cells and activate both CD4+ and CD8+ antigen-specific T effector cells is achieved for powerful antitumor effects in female C57/BL6 mice injected with EG7-OVA cells (mouse lymphoma cell line) (157, 158).

Gelatin is a natural versatile biopolymer; it has several important applications due to its low cost, biodegradable and biocompatible nature as well as the presence of abundant active groups (159). Gelatin nanoparticles show potential in terms of drug delivery due to excellent characteristics and can be degraded by gelatinases such matrix metalloproteinase (MMP) (160). Bu et al., developed a MMPs-degradable gelatin nanoparticles loaded with photosensitizer indocyanine green (ICG) along with signal transducer activator of transcription 3 (STAT3) inhibitor (NSC) for efficient photothermal therapy and immunotherapy of HNSCC. In the tumor tissue, gelatin nanoparticle was degraded and encapsulated ICG and NSC were effectively released. Under near-infrared irradiation, the released ICG nanoparticles enabled effective photothermal destruction of tumors, and the STAT3 inhibitor NSC elicited potent antitumor immunity for enhanced cancer therapy. The population of PD-1 cells presented a remarkable decrease compared to PBS control group after treatment with Gel-N-ICG NPs exposed to laser irradiation. Gel-N-ICG NPs with laser irradiation was demonstrated to have the ability to inhibit the immunosuppression of tumor microenvironment (TME), which can enhance the anti-tumor efficacy (161). In their work, Phung et al, present a modifying PLGA-PEG nanoparticle with Folic-Acid carrying a miR-200c inhibitor for PD-L1 expression. Their data showed increased accumulation of these nanoparticles inside TME *in vivo*, PD-L1 inhibition *via* microRNAs and the induction of a more immunogenic tumor microenvironment, also exhibiting increased dendritic cells maturation and CD8+ T cell response towards cancer cells (162). Another core-shell nanostructure composed of Calcium-Phosphate (CaP) dendrimer was developed as a dual-targeted therapy using a small interfering RNA (siRNA) against immune checkpoint ligand PD-L1 and the plasmid DNA (pDNA) encoding immunostimulatory cytokine IL-2 to modulate the TME and activate immune effector cells for Hepatocellular Carcinoma (HCC) treatment. The results showed increased tumor-infiltrating CD8+ T cells, high levels of secreted IL-2 in the TME and enhanced proliferation of tumor-specific cytotoxic T cells improving immunity and facilitating infiltration of activated T cells into HCC tumors (163).

Nanotechnology can also be applied *ex vivo* with utility in activating and expanding T cells prior to their adoptive T cell transfer for cancer immunotherapy. To improve *ex vivo* expansion of antigen-specific T cells, Guasch et al., studied the influence of a polymeric polyethylene glycol (PEG) hydrogel cross-linked with two fibronectin-derived peptides, cyclic Arg-Gly-Asp (cRGD) and cyclic Leu-Asp-Val (cLDV) to stimulate T cells prior to adoptive transfer (164). The hydrogels were decorated with a quasi-hexagonal array of gold nanoparticles functionalized with the activating antibody CD3 to initiate T-cell activation. Both cLDV and cRGD hydrogels showed higher T-cell activation (CD69 expression and IL-2 secretion) (164). Polymeric nanocarriers nanoparticles can also mediate *ex vivo* mRNA delivery to edit the genome of T-cells prior to adoptive transfer. These nanoparticles can be designed to target a particular cell subtype and, upon binding to them, stimulate receptor-mediated endocytosis, thereby introducing the synthetic mRNA they carry which the cells can now express. The data demonstrated that nanocarriers efficiently delivered mRNAs that encode a genome-editing agent that could efficiently eliminate selected genes in anticancer T cells and improve antitumor activities in T cells. Moreover, transfection with mRNAs that encode a key transcription factor of memory formation engineered nanoparticles has been shown to influence CAR-T cells toward a core memory phenotype. Overall, they demonstrated that a properly designed mRNA nanocarrier can perform controlled delivery of functional macromolecules to lymphocytes or hematopoietic stem cells by simply mixing of the reagent with the cells *in vitro* (165). A different approach, artificial antigen presenting cells (aAPCs) can be generated by coupling a major histocompatibility complex (MHC)–immunoglobulin (Ig) dimer (signal 1) and a co-stimulatory CD28 antibody (signal 2) to an iron-dextran nanoparticle (166, 167). The T cells activated by nano-aAPC in a magnetic field inhibited growth of B16 melanoma, showing that this novel approach, using magnetic field-enhanced nano-aAPC stimulation, can generate large numbers of activated antigen-specific T cells and has clinical relevance and applications for adoptive immunotherapy (167).

Nanoparticles have also been proposed as vaccine candidates (168). Nanovaccine can not only co-deliver tumor antigens and adjuvants to lymphoid tissues in close proximity, but also further enhance therapeutic efficacy by loading with immunosuppressive inhibitors or immunostimulatory compounds (169, 170). Gan et al, propose a lymph node targeting cancer vaccine by using CpG-loaded aluminum phosphate nanoparticles with a mouse cell membrane. After mice immunization, they observed strong cellular immunity, including potent IFN-γ+CD4+ T cells, IFN-γ+CD8+ T cells, cytotoxic T lymphocytes and cytokine excretion in spleen and lymph node cells leading to significantly tumor growth suppression and prolonged survival of mice in melanoma models (166). This vaccine delivery system shows great potential and can be further developed for personalized HNSCC cancer vaccines. Another approach used anti-CTLA-4 siRNA-loaded chitosan-lactate (CL) nanoparticles to facilitate priming anti- tumor T cells and the downregulation of CTLA-4 on tumor-infiltrating T cells were observed, which was associated with tumor regression and increased survival in a mouse tumor model. The effect was achieved through the reduction of immunosuppressive cells, the improved cytotoxicity of T lymphocytes, decreased inhibitory and increased inflammatory cytokines, and reduced angiogenesis and metastasis processes (167). The response rates of HNSCC to checkpoint blockade are below 20%, to increase its efficacy Tan et al., engineered a tumor antigen-targeted nanosatellite vehicle to enhance the efficacy of STING (stimulator of interferon genes) agonist and sensitize SOX2-expressing HNSCC to checkpoint blockade (148). IFN-I target genes include several Th1 chemokines, which are critical for the tumor-homing of APC and Tcell effectors (149). The combination of nanosatellite vaccine with anti-PD-L1 not only promotes CD8+ CTL but also reduces CTL exhaustion, delivering superior protection (148).In HNSCC, specific antigens such as HR-HPV oncogenic proteins, p53 and CSC-related proteins can prime immune cells to induce a robust immune responses (169, 171). It has been shown that Liposomes can be utilized to design therapeutic HR-HPV vaccine. A liposomal HPV16 mRNA formulation (HPV16 E7 RNA-LPX vaccine) was administered intravenously in murine HR-HPV16-positive and displayed a robust E7 antigen-specific CD8^+^ T cell response with a strong and sustainable memory phenotype. HPV-positive tumors of immunized mice were heavily infiltrated with activated immune cells and HPV16-specific T cells and were polarized towards a proinflammatory, cytotoxic and less immune-suppressive microenvironment (172). In addition, the combination of a PD-L1 with the HPV16 E7 RNA-LPX vaccine resulted in synergistic inhibition of tumor growth and significant survival benefit (172).

**Figure 5:**
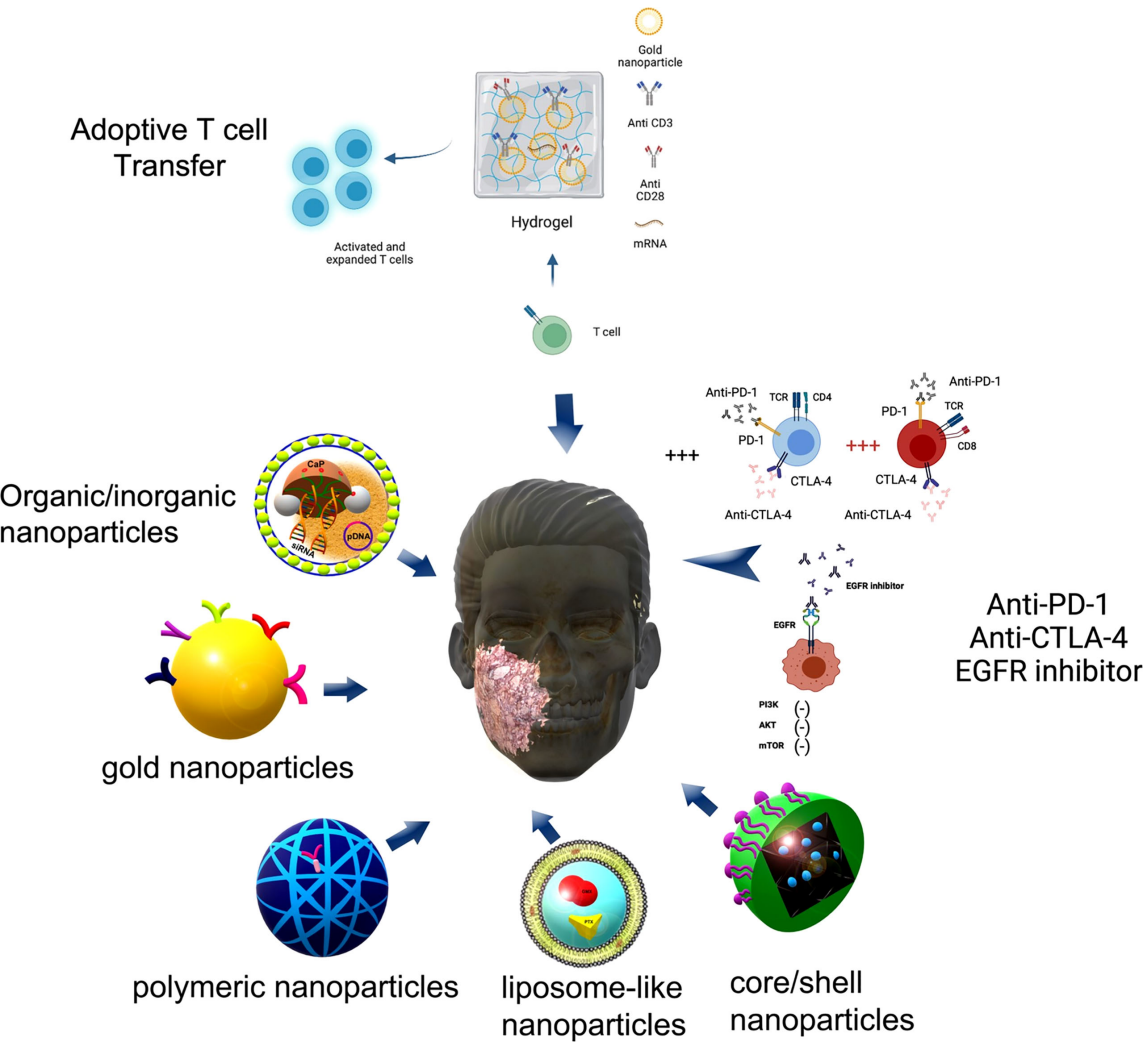
Nanoparticles proposed for immunotherapy. Some nanoparticles proposed for immunotherapy are pointed out here comprising organic/inorganic nanoparticles composed by lipid membranes and Calcium-Phosphate (CaP) for dual-targeted therapy using a small interfering RNA (siRNA) against immune checkpoint ligand PD-L1 and the plasmid DNA (pDNA) encoding immunostimulatory cytokine IL-2. Gold nanoparticles coated with antibodies such as cetuximab inhibit the proliferative downstream pathway PI3K/Akt/mTOR axis (—) due to EGFR blocking. Polymeric nanoparticles and core/shell nanoparticles can carry an anti-CTLA-4 siRNA or anti PD-1 antibodies increasing the percentage (+++) of effector CD4+ T cells and CD8+ T cells. Liposome-like nanoparticles carry immunotherapy drugs such as Gemcitabine (GEM) and Paclitaxel (PTX) working as drug delivery system. Figure generated using Biorender (https://biorender.com).

Mucin 1 (MUC1), a transmembrane glycoprotein, has shown to be as the possible prognostic marker to predict the risk of aggressive HNSCC (173). Radioresistance and radiosensitivity were also observed in HNSCC cells that are MUC1 overexpression and MUC1 under expression (174). Lipid/calcium/phosphate (LCP) nanoparticles modified with mannose were developed to deliver mRNA encoding MUC1 to DCs in the lymph nodes. The anti-CTLA-4 monoclonal antibody was combined with the mRNA vaccine to enhance the anti-tumor immune response by targeting regulatory pathways in T cells (175). *In vivo* studies demonstrated that the NP vaccine could induce a strong, antigen-specific, *in vivo* cytotoxic T lymphocyte response against 4T1 breast cancer cells; and that combination immunotherapy of the vaccine and anti-CTLA-4 monoclonal antibody could significantly enhance anti-tumor immune response compared to the vaccine or monoclonal antibody alone (175). Despite breast cancer being used in this study, the results can be employed similarly in future studies, since MUC1 can be a target for CAR‐T therapy in HNSCC (126). In the same context, Luo et al., developed a minimalist nanovaccine, comprising a simple physical mixture of an antigen and a synthetic polymeric nanoparticle, PC7A NP (176). The nanovaccine led to potent tumor growth inhibition in melanoma, colon cancer and human papilloma virus-E6/E7 tumor models. PC7A NP improves antigen delivery and cross-presentation in APCs and stimulates CD8 T cell responses. The combination of the PC7A nanovaccine and an anti-PD-1 antibody showed great synergy, with increase survival rate in animal tumor models; tumor growth was completely inhibited when these vaccinated animals, suggesting generation of antitumor memory (176). In order to study the effect of vaccine-induced immunologic targeting on the progression of viral-associated HNSCC, single (gp100) and multiple (B16-tumor lysate containing gp100) immunogenic viral antigens were encapsulated within differing molecular weight poly (lactic-co-glycolic acid) (PLGA) nanoparticles (177). The study reports differences in immunological potency attributable to alteration of polymer and the results showed that 80 KDa polymer was associated with greatest production of anti-tumor inflammatory/Th1 cytokines implying superior antigen cross-presentation. Moreover, the NP-mediated antigen delivery stimulated the production of immune-stimulating cytokines such as IFN-γ and reduced the production of immune-inhibitory cytokines such as IL-10 compared to the use of soluble tumor cell lysate (177). In another study, nanosatellites of iron oxide core conjugated with cGAMP and HPV16 E6/E7 peptides were developed to vaccination and to prevent HNSCC immune escape. They showed that the E6/E7-targeted nanosatellite vaccine expands the tumor-specific CD8+ T cells by over 12-fold in the tumor microenvironment and reduces tumor burden (178). A combination of nanosatellite vaccine with anti-PD-L1 significantly expands cytotoxic T lymphocytes tumor-specific and limits the populations expressing markers for exhaustion, resulting in more effective tumor control and improved survival (178).Mesoporous silica rods (MSRs) based vaccines were utilized to demonstrate the impact of immunogenic viral antigens on anti-tumor response and immune editing in MOC2-E6E7, a preclinical model of HNSCC which expresses HPV-16 E6 and E7 oncoproteins. Injectable MSR-vaccines were able to generate an E7-specific response in MOC2-E6E7 tumor-bearing mice treated with PBS alone or vaccinated with the E7 peptide-loaded MSR vaccine (179). The vaccination induced robust infiltration of antigen-specific CD8^+^ T cells, which led to tumor growth delay and modestly prolonged survival in HPV^+^ oral tumor mice mode l (179). **Figure 5** summarizes some applications of nanoparticles for immunotherapy.

## Conclusion

Elucidating HNSCC pathology, mechanisms of immune evasion and the immune response is ideal to develop new therapies and achieve better prognosis and survival. As discussed above, different components of the T cell immunology are relevant to HNSCC at least, the frequency of regulatory T cells plays a pivotal role since those cells increased in HNSCC suppress the immune system, regulating their suppressive function as well as their differentiation can cooperate with a better inflammatory response to target the tumor cells. On the other hand, effector T cells are in an unresponsive state due to prolonged antigen exposure. In this case, identifying genes that regulate T cell exhaustion and interfering with their expression to avoid and possibly rescuing the cells from the unresponsive state can be another strategy as well as the regulation of T cell function and migration could help directly specific T cell response to the tumor microenvironment. Moreover, understanding the tumor microenvironment can contribute to identifying specific T cell populations to be targeted and stratify patients as low and high risk for HNSCC to use therapies accordingly to patients’ immune signature. Patients who present high frequency of regulatory T cells in the tumor microenvironment can benefit from the specific inhibition of those cells. Patients that present high frequency of cells expressing inhibitory molecules such as PD-1 and CTLA-4 can benefit from the use of inhibitors, but as previously discussed, the evaluation of specificity versus efficacy of those inhibitors need to be considered. By understanding how over or under expression of HNSCC mutated genes upregulate key proteins related to proliferation, survival and apoptosis associated with immune evasion mechanisms can aid the development of tailored strategies for individualized treatment. Targeting mutated genes and/or kinases should be considered as option instead of blocking interaction between inhibitory molecules and their ligands in order to avoid side effects and also lead to better efficiency. In this case, the use of phosphatase inhibitors could be an option for patients who the use of PD-1 inhibitor has not been efficient enough. Similar approach can be applied or evaluated for different T cells, distinct inhibitory molecules and gene mutated, depending on the patients’ immune profile. The safety, specificity and efficiency of those new approaches should also be evaluated. New therapies described in this review such as the use of nanoparticles and the CAR T cells bring to reality the possibilities of more efficiency, better outcome, and less toxicity in cancer treatment. Moreover, individualized immunotherapies should be considered due to patient genetic differences, distinct immune response, and tumour phenotypes. The evaluation of combined therapeutic approaches to block inhibitory responses and at the same time induce effector response should be better discussed in literature. In summary, improved therapeutic strategies to treat HNSCC are necessary, and data published on the use of nanoparticles and CAR T cells to treat cancer are promising. However, further investigation involving those therapies as well as other cancer therapies is required prior their usage in patient care to identify the safest/most efficacious therapeutic protocols.

## Author contributions

Design and Conceptualization: MD, CN, LA, VO, and CC-S; Literature Revision: MD, CN, and VO; Writing of the manuscript: MD, CN, LA, VO, and CC-S; Supervision: LA and CC-S; Funding Acquisition: LA and CC-S. All authors contributed to the article and approved the submitted version.

## Funding

This study was partially supported by the Coordenação de Aperfeiçoamento de Pessoal de NívelSuperior – CAPES - Finance Code 001 and FAPEMIG – Rede Mineira de Nanomedicina Teranóstica (RED-00079-22).

## Conflict of interest

The authors declare that the research was conducted in the absence of any commercial or financial relationships that could be construed as a potential conflict of interest.

## Publisher’s note

All claims expressed in this article are solely those of the authors and do not necessarily represent those of their affiliated organizations, or those of the publisher, the editors and the reviewers. Any product that may be evaluated in this article, or claim that may be made by its manufacturer, is not guaranteed or endorsed by the publisher.
